# The Origin of Defective Enamel

**Published:** 1884-09

**Authors:** W. H. Eames

**Affiliations:** St. Louis, Mo.


AMERICAN JOURNAL

OF

DENTAL SCIENCE.

VOL. XVIII. THIRD SERIES—SEPTEMB'R, 1884. No. 5.

ARTICLE I.

### THE ORIGIN OF DEFECTIVE ENAMEL.

BY W. H. EAMES, D. D. S., OF ST. LOUIS, MO.

(Read before the Illinois State Dental Society.)

At your last annual meeting I had the pleasure of presenting before this Society a short introductory paper on the subject of defective enamel structure. It embraced a synopsis of the views of the most prominent writers upon the subject, together with some objections which have presented themselves to my mind in the study and investigation of this question. Time did not permit a full confession of the subject, and now, in fulfilment of a promise made to the Society, I bring the subject before you again in the following brief paper, in which I have endeavored more fully to present my views.

The theories entertained by all writers of the present day, as to the cause or origin of defective enamel structure, differ but little, if any, from their predecessors, writing perhaps a century earlier. It seems to be regarded as one of the settled facts of our science, which, like the traditions of the Church, it were heresey to dispute; it is set forth in all our text books, and taught in all our colleges; but notwithstanding the general acceptance which it has met at the hands of all succeeding students, it seems to me, that whether it is capable of disproof or not, there is reasonable room for doubt at least—and while I do not expect to present a solution of the phenomena of these various formations which shall be accepted as true without question, I do hope to illumine some of the darker places in the misty theories of the past.

The various forms of defective enamel which, up to the present time, have been knows as rocky, ridged, furrowed, cribriform, pitted and grooved enamel, attributed to one cause, viz., arrested development, will be considered under two general heads, "Congenital" and "Non-Congenital" or accidental. The first class, "Congenital," will include all of those defects which are supposed to originate during the formative period of the tissue; the second class, (non-congenital or accidental,) those forms which originate after the complete development of the tissue.

Thus the first class, or congenital, will comprise some of those forms known and described as rocky or ridged enamel; simple pits in the structure more commonly found on the points of the cusps, but existing often on other portions, of the tooth, and especially noticeable in cases where but few of the teeth are affected: grooves or fissure in the coronal surfaces of the bicuspids and molars. The second class (non-congenital or accidental) will include some of the forms of grooved or furrowed teeth not considered in class first, described by Mr. Hutchinson and others as due to syphilis, mercury, etc.; also, cases presenting the same characteristics which occur later in life, in the form of grooves at the gingival border of the molars and bicuspids, usually considered as cases of erosion, abrasion, denudation, atrophy and caries, which, in my opinion, have a common origin.

The origin of the first class, as I believe and hope to demonstrate, is not due to constitutional disturbance affecting the epithelial structures, such as measles, eruptive fevers, mercury, syphilis, eclampsia, etc., which are supposed to cause an arrest of development and a consequent defective enamel structure, but to a defect in the formative organ, which might, in cases of pits, be termed a blight or death of the ameloblast, or in cases of fissures a "rupture," being the result of a separation of the ameloblastic layer.

Let us glance at the process of development at this period. According to the best and latest authority on this subject the enamel organ envelopes the pulp or dentine organ in the form of a hood, corresponding somewhat to the form of the future tooth. It is composed of three layers, or strata: an external and an internal epithelial layer, and a reticular layer; the external and internal layers are formed of the columnar cells of the malpighian layers of the epithelium. The internal layer of cells or ameloblasts, having undergone a change in length, are much longer than the cells composing the external layer. To the cells composing the internal layer, I have given the name "*ameloblasts*," as to them is delegated the work of forming the dentine. Just how this process of amelification takes place is not definitely determined. Whether it be by direct impregnation of the cell or by exudation, is not essential, so long as it be admitted that the formation of the enamel is dependent on the presence of the ameloblasts. The recent explanations of the phenomena of enamel formation, by Prof. Bodecker, Williams and others, if true, might affect the position taken in this paper, but being in advance of the accepted theories up to the present day they will not be considered.

There is no class of cells more liable to a change of a retrogressive character than the epithelial, and none develop more rapidly. We have seen that the enamel organ is made up of this class of cells exclusively, and is therefore subject to changes noticeable in epithelial structure elsewhere; a blight or death of one or more of the cells of this organ may be looked for at any time. If one or more of the ameloblasts die, or from any cause be wanting in the internal layer of the enamel organ at any point, then will there be a missing rod or rods corresponding in number with the missing ameloblasts, thus forming a circular pit. If the ameoblasts were missing when the process of amelification commenced, this circular pit would extend from the external surface of the enamel to the surface of the dentine beneath; but if the cells die after the process of amelification is partially completed, then the depth of the pit will correspond to the length of the rods which were left unfinished; the cells surrounding the missing ones being unaffected would complete the work assigned to them, and thus the walls of this pit will be normal in character, except, perhaps, in cases where the direction of the rods is slightly changed. Thus is formed the pit described by Wedl and others, "an opening surrounded by enamel rods and the bottom clothed with enamel."

The change which takes place in the walls of these pits from the action of external agents, acids, bacteria, etc., after the tooth is erupted, would result in the forms figured by Wedl and Magitot, and by them described as being filled with pigment, debris of tooth substances, food, leptothrix, etc. The breaking up of the rods forming the wall, giving the pit the form of a pouch or cul-de-sac, may be accounted for in this manner. It may be claimed that this would be but a form of arrested development, especially as this process of amelification is one step in the process of development; but can it be called an arrest of development "dependent upon some constitutional disturbance," as Wedl, Magitot, Tomes and others assert? We think not. It is not an arrest, but a death or blight of a cell or cells forming the enamel rods, independent of any systemic or general constitutional disturbance.

The next form to which I would call attention, fissures or grooves in the coronal surfaces of molars and bicuspids, is also accounted an arrest of development, a lack of coalescence, a failure on the part of the enamel plate, which, in its development from the cusps towards the center, fails to meet and coalesce. To Dr. Black, of Jacksonville, belongs the credit of the following solution of this defect, which, evidently, is not due to a failure to coalesce, but is rather the result of a rupture of the enamel organ at this point, a separation of the ameloblastic layer, thus separating the rods and forming a fissure.

At the period of development, when this rupture takes place, the pulp has assumed the form of the future tooth, but not its full size. Small caps of dentine are formed over the points of the cusps; the enamel organ has undergone the change noticeable in the form of the cells, and a change is taking place, noticed by Mr. Tomes and others, namely, changing from protoplasm to formed material, ready to receive the lime-salts. This change, which renders the cells stiff and unyielding, commences directly over the points of the cusps and passes gradually out from these points, as centers, towards the center of the grinding surface, and down towards the sides of the crown. The growth of the dental bulb stretches the investing enamel organ, and the points over the cusps being fixed, the strain comes directly on the softest and most yielding portion, midway between the cusps, where fissures are to be found. The strain upon the enamel sheet separates the ameloblasts, and thus a break in their continuity occurs, which is not repaired, and a consequent fissure is formed in the sheet of enamel. The term coalition implies the union of two separate portions which are independent of each other; but we have seen that the enamel grows from a continuous sheet of ameloblasts, each connected with the other, so that here there can be no failure to coalesce. It is true that the deposit of lime-salts takes place from certain points, but the receptacles for the lime envelope the entire crown. These fissures are most frequently found on teeth having prominent cusps, as in such cases the enamel organ is most subject to rupture. These cracks or fissures which before the eruption of the tooth present smooth walls of enamel rods, soon after eruption become discolored and filled with debris and leptothrix, and assume the appearance as seen under the microscope and described by Wedl. This defect in the enamel is of the same origin as the form described as pits—the absence of the ameloblasts, differing only in the cause; and most of the defects of this character, no matter what forms they may assume or where located, have this origin in which special constitutional disturbances play no part.

Of the second class of cases of defective enamel, the non-congenital or accidental as was suggested in my paper of last year, I believe a large number to be due to the abnormal action of the so-called absorbent organ. At the time when some of these defects occur the temporary teeth occupy their sockets in the alveoli; the permanent teeth are inclosed in the follicular wall, surrounded by a bony crypt, which, with the roots of the temporary teeth, must be removed to allow them to assume their position in the arch. Nature has provided an agent to do this work. Mr. Tomes designates it as the absorbent organ, composed of a peculiar class of cells resembling the myeloid cells of Kolliker. Wedl designates these cells as *osteo, or odontoclasts*. We believe them to be simply granulation cells. This organ, whatever it be, is found in close contact with the tissue about to be removed, whether bone or tooth. Cup-shaped excavations filled with this cellular organ are noticeable on the surfaces being acted upon. The removal of the roots of the temporary teeth and the opening of the crypts for the eruption of the permanent teeth is said to be a physiological process, and yet it is so nearly allied in all physical characteristics to the other processes, termed pathological, that it is difficult to distinguish between them, so as to be able to say what characteristics mark a physiological, and what a pathological process. We are of the opinion that the processes are identical, differing only in the circumstances producing them.

Again, it is stated that the method employed by nature for the removal of the temporary teeth is *sui-generis*. We must dissent from this view, believing it to be the same process that she has provided for the removal of other tissues.

The removal of the protruding stump of a limb, by the granulating cells, after the sloughing off of the soft parts, is identical in form and results, with the removal of the temporary teeth. It is simply a result following one of the phases of inflammation ; the result of an excitation in a part, otherwise normal, inducing a proliferation of cell growth, which may be progressive or retrogressive in character, or both interchangeable; at one time acting as an absorbing organ, and at another as a builder up of tissue, dependent, as is generally supposed, on the amount of life-force present.

Let us apply this view of the character and action of the so-called absorbent organ to the removal of the roots of the temporary teeth. When the crown of a permanent tooth is completed and the growth of the tooth commences (if the process goes on normally,) the growth of the root forces the crown onward against the hard tissue, covering it with force sufficient to produce excitation and promote the proliferation of cell growth in the part ; and these cells, so far as we are able to learn, are identical in character with those found in a granulating wound and produced under similar conditions, the same as are produced in cases of inflammation of a low type, which result in destruction of tissue.

Here then we find the development of the absorbent cells in the part ready to remove any obstruction to the continued growth of the permanent tooth. Thus is the crypt of the permanent tooth, the alveolus and roots of the temporary rooth removed. It frequently happens that the partial removal of a temporary root is sufficient to relieve the pressure produced by the incoming tooth, changing the cells proliferated from a retrogressive character ; and the process of building up of tissue succeeds—and this again may be succeeded by absorption.

It is a noticeable fact that the roots of replanted teeth are frequently removed by a process identical, in all respects, to the removal of the temporary teeth. This occurs, in some cases, soon after the operation of replantation; in other cases several years may elapse before the tooth is lost. Smoetimes this work of removal commences at the apex of the root, at other times it will be noticed at the gingival border.

The removal, by nature, of the roots of replanted teeth is termed a pathological process; and yet wherein does it differ from the removal of the roots of the temporory teeth, which is recognized as a physiological process? It is, undoubtedly, one and the same process, dependent on the same causes for its action, and governed and controlled by the same laws. The conditions in the case of a replanted tooth are eminently fitted to induce this change, causing a proliferation of cell growth; the circulation in the parts surrounding the root being defective, a low type of inflammation is readily set up, and the development of cells of a retrogressive character may reasonably be looked for, sooner or later, in every case.

Just how these cells do their work is as yet an unsettled question; most writers inclining to the opinion that a fluid of exudation is present which dissolves out the lime-salts from the hard tissues with which it comes in contact. The following brief extract, from a letter by Dr. G. V. Black, Jacksonville, Ills., on "Ferment," is the best explanation of the action of the absorbent organ, and is, in my opinion, in advance of anything which has been written on this subject: "Absorptive and resorptive digestion is accomplished by a soluble ferment, eleborated by the tissues under special circumstances, for the removal of tissues no longer needed as roots of temporary teeth. The absorption of the temporary teeth is a physiological process, as all other absorptive processes are. It is brought about by the action of a class of cells which have become known as 'osteo' or 'odontoclasts.' These are only the oridinary connective tissue cells of the part in immediate proximity with the part to be removed, which have temporarily taken on new functions—the secretion or eleboration of a soluble ferment for the removal of these roots of the temporary teeth which are no longer wanted. These cells perform this act in an indirect manner. It is plain that these roots are not removed by any mere mechanical force. These cells have no physical power of growing into them ; they secret a soluble ferment, analogous to the body which digests them, breaks them down and fits their substance for entering into the blood streams by osmosis, just as solid ingesta in the stomach is broken down and *fitted* to enter the circulation by osmosis.

"There seems to be no foundation for the notion that the resorption of these roots may not form proper pabulum for the building up of other tissues ; that the absorbed product is necessarily excreted. From accident or decay the temporary teeth often lose their pulps, which sometimes results in the formation of an alveolar abscess. If such an abscess exists at the time absorption should take place, absorption fails, partially or entirely ; the tissue which performs this function being thrown into a pathological state or condition, the secretion of the soluble ferment does not take place in the usual manner.

" If, however, the abscess does not occur, or if such abscess be cured and the tissues in immediate proximity to the devitalized root be perfectly healthy, it is found that the absorption goes on in the usual manner. The mere death of the root does not interfere with the resorption, provided the physiological condition is maintained in the immediately surrounding cells, which are the active agents in the work. The soluble ferment does not depend for its action upon the life of the tissue to be acted upon. The roots are simply digested and enter the blood streams by osmosis, as any other digested material."

Many of the forms of honey-combed and furrowed teeth (incisors and six-year molars) closely resemble, in general appearance, surfaces of hard tissue which have been acted upon by the absorbent organ ; incisors, marked by horizontal ridges and furrows, are said to have their origin in some constitutional defect, causing an arrest of develepment, in the epithelial structure—the furrows indicating a period of arrest, the ridges a period of development, thus forcing the conclusion that there has been a succession of disturbance equal in number to the markings on the teeth. This can hardly be accepted as true, especially in cases where no constitutional disturbances have been observed. May not much of this marking on the incisors and sixth-year molars be due to the action of the absorbent cells? Is it not possible that, having performed their work of removing the temporary teeth and the alveoli, they continue to act and remove the enamel cuticle and dissolve out the lime from the freshly amelified enamel rods with which they come in immediately contact at the gingival border?

The succession of furrows observed, may be accounted for by assuming that there is a period of growth and a period of rest for the incoming tooth; the cells, acting upon the enamel when the tooth is at a period of rest, form a furrow or groove across the surface. When the period of growth sets in, the tooth shoots onward, and a portion of unaffected, perfect enamel passes beyond the reach of their influence, forming a ridge; again a period of rest sets in, and again the cells act upon the enamel brought in immediate contact, and another furrow is formed; thus any number of furrows and ridges are formed, dependent upon the continuance of the abnormal action of these cells. As this action is due to systemic conditions, all teeth in contact with the organ at the time are alike acted upon; hence the relative position of the markings on the incisors and canine teeth.

The sixth-year molar is acted upon in a similar manner by the organ which lies directly over and in contact with the grinding surface. The surface presents a series of pits and cup-shaped excavations, reaching, at some points, the dentine beneath. Spines of perfect, unaffected enamel, the full depth of this tissue at this point, are frequently seen surrounded by these pits and excavations; the change noticeable in color, and the rounding up of the otherwise sharply outlined borders of these excavations, is due to subsequent action. The cases known as erosion, occuring at the gingival border, are in many instances, undoubtedly the result of this same action of the granulation cells. What the conditions are which contribute to this abnormal action are, as yet, a hidden mystery. Like many other afflictions to which flesh is heir, we know not the cause thereof.

Touching the action of these cells, I quote another paragraph from Dr. Black's lecture on "Ferments," which may, in somewise tend to clear our minds on this subject: "We have reasons to suspect, at least, that soluble ferments are often found in places where they are not wanted. Tissues are stimulated to false secretions by various irritants and in many ways.

It is probably that many of the excriating secretions, often met with in children, are soluble ferments given out through some mal-condition of the tissues. We have reason to suspect that, in some, decay of the teeth may come about in this way. We have already seen that tooth substance is digested and removed by a soluble ferment normally formed; the same is possible in an abnormal way. We have often noticed a peculiar irritated condition of the gums about the necks of the teeth, especially the inferior incisors, and that decay occurs in these portions exposed to the secretions thrown out. Twelve years ago, long before the conception of the ideas inculcated in this paper, we called attention to the connection between this irritation and decay, in a paper before this society. Irritation from a clasp-plate forms cavities for lodgment of colonies of bacteria.

Drs. Underwood and Miles, of London, claim to have found a form of growth in the mouth which grew directly into the surface of the teeth, otherwise perfect. Dr. Miller, of Berlin, seems nonplussed by the fact that he finds the softening of dentine in caries always in advance of the organisms. This is exactly what we should expect to find; for if decay is produced by the organisms, it must be by the digestive fluid, soluble ferments thrown out by them, or by their waste of products. When Dr. Koch was examining the gangrene, produced in mice by the coco-bacterium of hospital gangrene, he found uniformly that the tissues were destroyed in advance of the bacteria. The bacteria seemed not to touch the living tissues at any time after the work was fairly begun, the tissues being destroyed in advance by the soluble ferment."

[TO BE CONTINUED.]

